# Assessing performance of pathogenicity predictors using clinically relevant variant datasets

**DOI:** 10.1136/jmedgenet-2020-107003

**Published:** 2020-08-25

**Authors:** Adam C Gunning, Verity Fryer, James Fasham, Andrew H Crosby, Sian Ellard, Emma L Baple, Caroline F Wright

**Affiliations:** 1 College of Medicine and Health, University of Exeter Medical School Institute of Biomedical and Clinical Science, Exeter, Devon, UK; 2 Exeter Genomics Laboratory, Royal Devon & Exeter NHS Foundation Trust, Exeter, UK

**Keywords:** genetics, genetic testing, genetic variation, genomics, human genetics

## Abstract

**Background:**

Pathogenicity predictors are integral to genomic variant interpretation but, despite their widespread usage, an independent validation of performance using a clinically relevant dataset has not been undertaken.

**Methods:**

We derive two validation datasets: an ‘open’ dataset containing variants extracted from publicly available databases, similar to those commonly applied in previous benchmarking exercises, and a ‘clinically representative’ dataset containing variants identified through research/diagnostic exome and panel sequencing. Using these datasets, we evaluate the performance of three recent meta-predictors, REVEL, GAVIN and ClinPred, and compare their performance against two commonly used in silico tools, SIFT and PolyPhen-2.

**Results:**

Although the newer meta-predictors outperform the older tools, the performance of all pathogenicity predictors is substantially lower in the clinically representative dataset. Using our clinically relevant dataset, REVEL performed best with an area under the receiver operating characteristic curve of 0.82. Using a concordance-based approach based on a consensus of multiple tools reduces the performance due to both discordance between tools and false concordance where tools make common misclassification. Analysis of tool feature usage may give an insight into the tool performance and misclassification.

**Conclusion:**

Our results support the adoption of meta-predictors over traditional in silico tools, but do not support a consensus-based approach as in current practice.

## Introduction

As the scale of genomic sequencing continues to increase, the classification of rare genomic variants is the primary bottleneck in the diagnosis of rare monogenic disorder. Guidelines published by the American College of Medical Genetics (ACMG) in 2015^1^ have helped to bring greater consistency to variant classification. These have been followed by gene/disorder-specific rule-sets,^2 3^ and country/healthcare system–specific guidance such as the UK Association for Clinical Genomic Science (ACGS) best practice guidelines for variant interpretation.^4^ Common to all guidelines is the recommendation of the use of in silico prediction tools to aid in the classification of missense variants. In silico prediction tools are algorithms designed to predict the functional impact of variation, usually missense changes caused by single-nucleotide variants (SNVs). Though originally designed for the prioritisation of research variants,^2^ the tools are used routinely in clinical diagnostics during variant classification. The tools integrate a number of features in order to assess the impact of a variant on protein function.^3^ Initially, inter-species conservation formed the bulk of the predictions, with some additional functional information, such as substitution matrices of physicochemical distances of amino acids (such as Grantham^5^ or PAM^6^), and data derived from a limited number of available X-ray crystallographic structures.^7^ Since the development of the first in silico prediction tools over a decade ago,^2 7^ large-scale experiments such as the ENCODE project^8^ have generated huge amounts of functional data, and we now also have access to large-scale databases of clinical and neutral variation.^9–11^ These additional sources of data have led to an explosion of new in silico prediction algorithms^12–14^ that purport to increase accuracy.

However, the large increase in the number of predictors integrated into classification algorithms has raised concerns about overfitting.^15 16^ Overfitting occurs when the prediction algorithm is trained on superfluous data or features that are irrelevant to the prediction outcome.^16^ While it may appear that an increasingly large feature list leads to improvements in prediction, random variability within the training dataset may result in decreased accuracy when applied to a novel dataset. Overfitting can be mitigated through the use of increasingly large training datasets, and the usage of online variant databases, such as the genome aggregation database (gnomAD)^17^ and ClinVar,^10^ allows for sufficiently large training datasets. In addition, reliance on additional information—such as protein functional data and allele frequency data such as from gnomAD^17^—may be contrary to the standard assumptions of variant classification methodology, namely that each dataset is independent and applied only once during classification.

The 2015 ACMG guidelines recommend the use of a concordance-based approach, where several prediction algorithms are used, and evidence is applied only when there is agreement between tools. There is no guidance on which in silico tools should be used, how many or on what constitutes a consensus, and this ambiguity allows for inconsistencies in the application of this piece of evidence across clinical laboratories. Studies have previously identified the limitations of applying a strict binary consensus-based approach.^18^ In response, multiple groups^12–14^ have created meta-predictors; tools which integrate information from a large number of sources into a machine-learning algorithm. These tools thereby adhere to the principle of the consensus-based model suggested by ACMG without the onerous task of determining tool concordance and reduce discordance when increasingly large numbers of tools are used. Unlike a manual consensus-based model, where tools are weighted equally, meta-predictors can apply weighting to features in order to maximise accuracy. The UK ACGS guidelines^4^ suggest it is likely that a single meta-predictor will replace this concordance-based approach, but a comprehensive analysis using a clinically representative dataset has not yet been done.

In order to evaluate the accuracy of in silico prediction tools, precompiled variant datasets such as VariBench^19^ have been designed to aid in training and benchmarking of pathogenicity predictors. However, the use of standardised datasets may introduce inherent biases into prediction algorithms, resulting in overfitting and false concordance. Typically, prediction software is trained using machine-learning algorithms, and assessed using variants available from large online public databases^2 3 7 8 12–14 20^ such as ExAC/gnomAD, ClinVar^10^ and SwissProt.^21^ It has been previously shown that prediction algorithms have variable performance when applied to different datasets,^3 20 22 23^ and therefore the use of variant datasets derived from online public databases may not be representative of the performance of tools when applied in a clinical setting. While studies emphasise the use of ‘neutral’ variation, the output from a modern next-generation sequencing pipeline is generally far from neutral and includes a large number of variant filtering steps in order to reduce the burden of manual variant assessment.^24^

**Figure 1:**
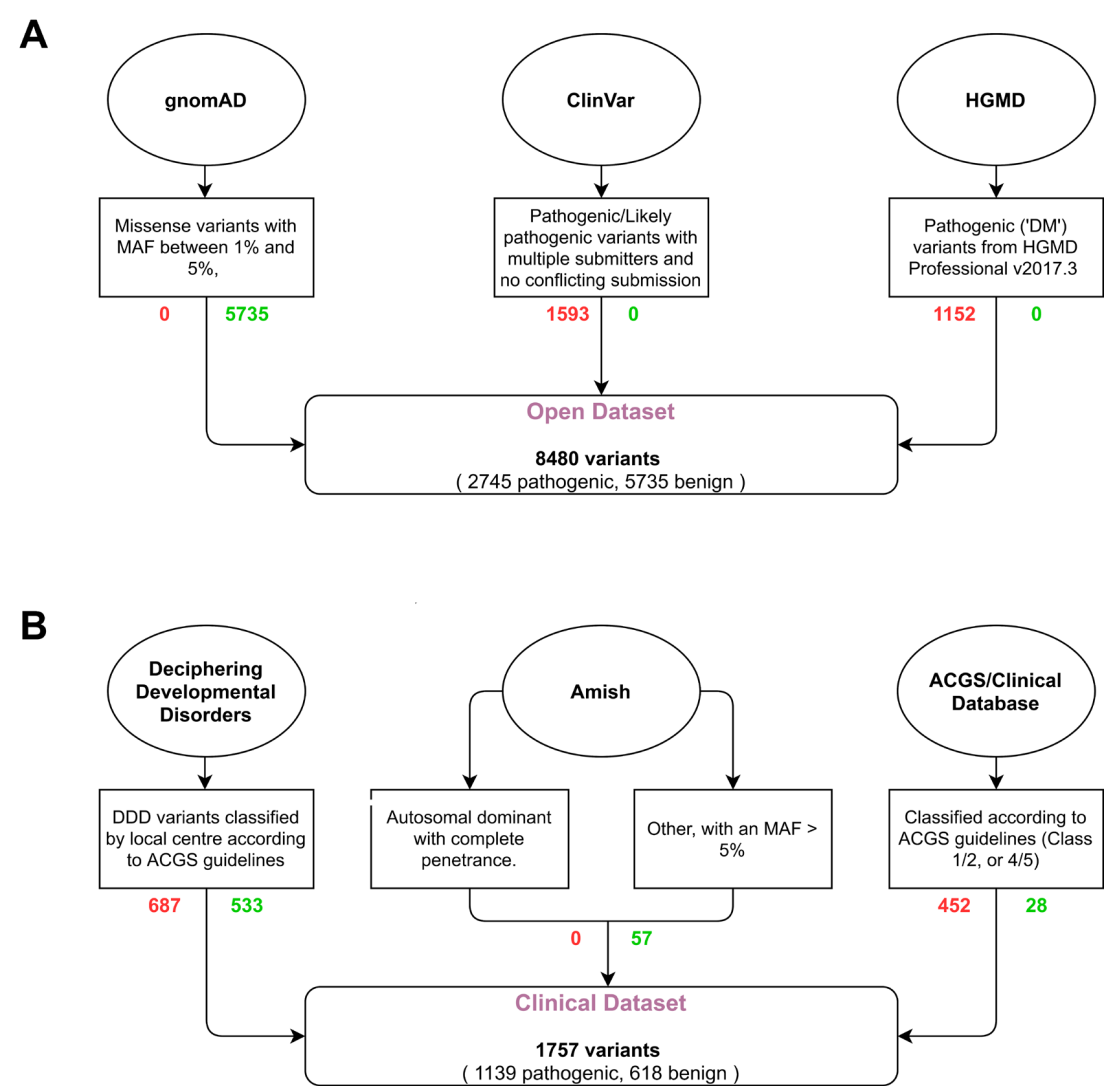
Flow diagram of selection and filtering steps used for the generation of the open (A) and clinical (B) datasets. Oval—variant source; box—selection criteria; rounded box—dataset. Red text (right) shows the number of pathogenic variants, green text (left) shows the number of benign variants. MAF, minor allele frequency.

Here, we evaluate and compare the performance of two traditional in silico pathogenicity prediction tools commonly used for clinical variant interpretation (SIFT^2^ and PolyPhen-2^7^), and three meta-predictors (REVEL,^12^ GAVIN^13^ and ClinPred^14^) using a publicly available (‘open’) variant dataset and a clinically relevant (‘clinical’) variant dataset (see figure 1). While a number of other tools are available, these metapredictors were selected as they were designed in the anticipation of being used in a clinical setting. We show that the tools’ performance is heavily affected by the test dataset, and that all tools may perform worse than expected when classifying novel missense variants. By assessing the effect of a consensus-based approach, our results support the use of a single classifier when performing variant classification.

## Materials and methods

### Open dataset

Open dataset (n=8480, see figure 1A) represents the typical training and validation dataset used during in silico predictor design and benchmarking. Positive (‘pathogenic’) variants were downloaded from ClinVar^10^ on 13 November 2017 and subscription-based HGMD^25^ Professional release 2017.3; neutral (‘benign’) variants in OMIM^26^ morbid genes were downloaded from the gnomAD^9^ database (exomes only data v2.0.1). *ClinVar criteria*: Stringent criteria were used to increase the likelihood of selected variants being truly pathogenic. Missense SNVs with either ‘pathogenic’ and/or ‘likely pathogenic’ classification, multiple submitters and no conflicting submissions were included; variants with any assertions of ‘uncertain’, ‘likely benign’ or ‘benign’ were excluded. *HGMD Pro criteria*: Single-nucleotide missense variants marked as disease-causing (‘DM’) were taken from HGMD Professional release 2017.3. *gnomAD criteria*: Missense SNVs with an overall minor allele frequency (MAF) between 1% and 5% were selected. These variants were deemed too common to be disease causing but are not necessarily filtered out by next-generation sequencing pipelines depending on the MAF thresholds used. Chromosomal locations with more than one variant (multiallelic sites) were excluded. Any variants found to be present in the ‘pathogenic’ and ‘neutral’ datasets were removed from both. Variants present in the SIFT, Polyphen-2, REVEL, GAVIN and ClinPred training datasets were removed to reduce bias and circularity. Variants with missing or intermediate scores were also removed.

### Clinical dataset

Clinical dataset (n=1757, see figure 1B and online supplementary table S1) more accurately reflects variants that might require classification in a clinical diagnostics laboratory following identification in an exome or genome sequencing pipeline. Variants were selected from three sources. *Group 1* (‘DDD’) consists of pathogenic (n=687) and benign (n=533) missense variants identified from 13 462 families in the Deciphering Developmental Disorders (DDD) study that have been through multiple rounds of variant filtering and clinical evaluation.^24 27^ Variants were identified through exome sequencing and were reported to the patients’ referring clinicians for interpretation and confirmation in accredited UK diagnostic laboratories. All benign variants from this list were assessed as having no contribution towards the patient’s phenotype, and were present in either as heterozygotes in monoallelic genes or homozygotes in biallelic genes classified according to the Developmental Disorder Genotype-2-Phenotype database (DDG2P)^28^ (data accessed 17 Oct 2019). *Group 2* (‘Diagnostic’) consisted of pathogenic (n=452) and benign (n=28) missense variants identified through Sanger sequencing, next-generation sequencing panel analysis or single gene testing in an accredited clinical diagnostic laboratory. Variants were manually classified according to the ACMG guidelines on variant interpretation^1^ on a 5-point scale (data accessed 23 Apr 2019). *Group 3* (‘Amish’) consisted of benign missense variants (n=57) identified through a Community Genomics research study of 220 Amish individuals. Variants were identified through singleton exome sequencing and were classified as benign based on population frequencies and zygosity within this study. Two subgroups were manually selected and annotated based on inheritance pattern and disease penetrance; subgroup (i) consisted of variants in genes that cause a dominantly inherited disorder with complete penetrance in childhood, for which the individual was clinically unaffected; this list was curated by a consultant in clinical genetics; subgroup (ii) consisted of variants in all other OMIM morbid genes (including those with incompletely penetrant dominant disorders and recessive and X-linked inheritance), with MAF >5% in the Amish cohort and MAF ≤0.01% in gnomAD (data accessed 18 Oct 2019). Variants with missing or intermediate scores were removed.

10.1136/jmedgenet-2020-107003.supp1 Supplementary data

### Transcript selection and variant annotation

For the open dataset, the canonical transcript was selected for each variant using the Variant Effect Predictor (VEP).^29^ For the clinical dataset, the HGMD Professional RefSeq transcript was used, unless absent from the database, in which case the MANE primary transcript was selected. Variants were annotated with variant cDNA and protein nomenclature in reference to the selected transcript. PolyPhen-2 and SIFT scores were annotated using VEP. REVEL and ClinPred scores were annotated using flat files containing precomputed scores for all possible single-nucleotide substitutions, and in both cases, the combination of nucleotide position, nucleotide change and amino acid change was sufficiently unique to identify a single record, that is, transcript selection did not affect the scores. GAVIN scores were generated through a batch submission to the GAVIN server.

### Tool benchmarking

The performance of each of the tools was determined for both datasets. For SIFT, PolyPhen-2, REVEL and ClinPred, the output of the analysis was a numerical score between 0 and 1. Initially, all tools were analysed according to the criteria defined in their original publications, with the thresholds for pathogenicity being ≤0.05 for SIFT, ≥0.9 for PolyPhen-2 and ≥0.5 for ClinPred. For REVEL, where no threshold is recommended, a threshold of ≥0.5 was used. The categorical classification of GAVIN was used directly (‘Benign’, ‘Pathogenic’). A supplementary analysis was done for those tools with a numerical output (SIFT, PolyPhen-2, REVEL and ClinPred) to more accurately compare their performance. A unique threshold was selected for each tool to calculate the specificity when sensitivity was set to 0.9. In order to include GAVIN in this analysis, a third analysis was performed, whereby each tool's specificity was measured when the threshold was adjusted to set the sensitivity identical to that of GAVIN.

## Results

### Classification of variant sources

**Figure 2:**
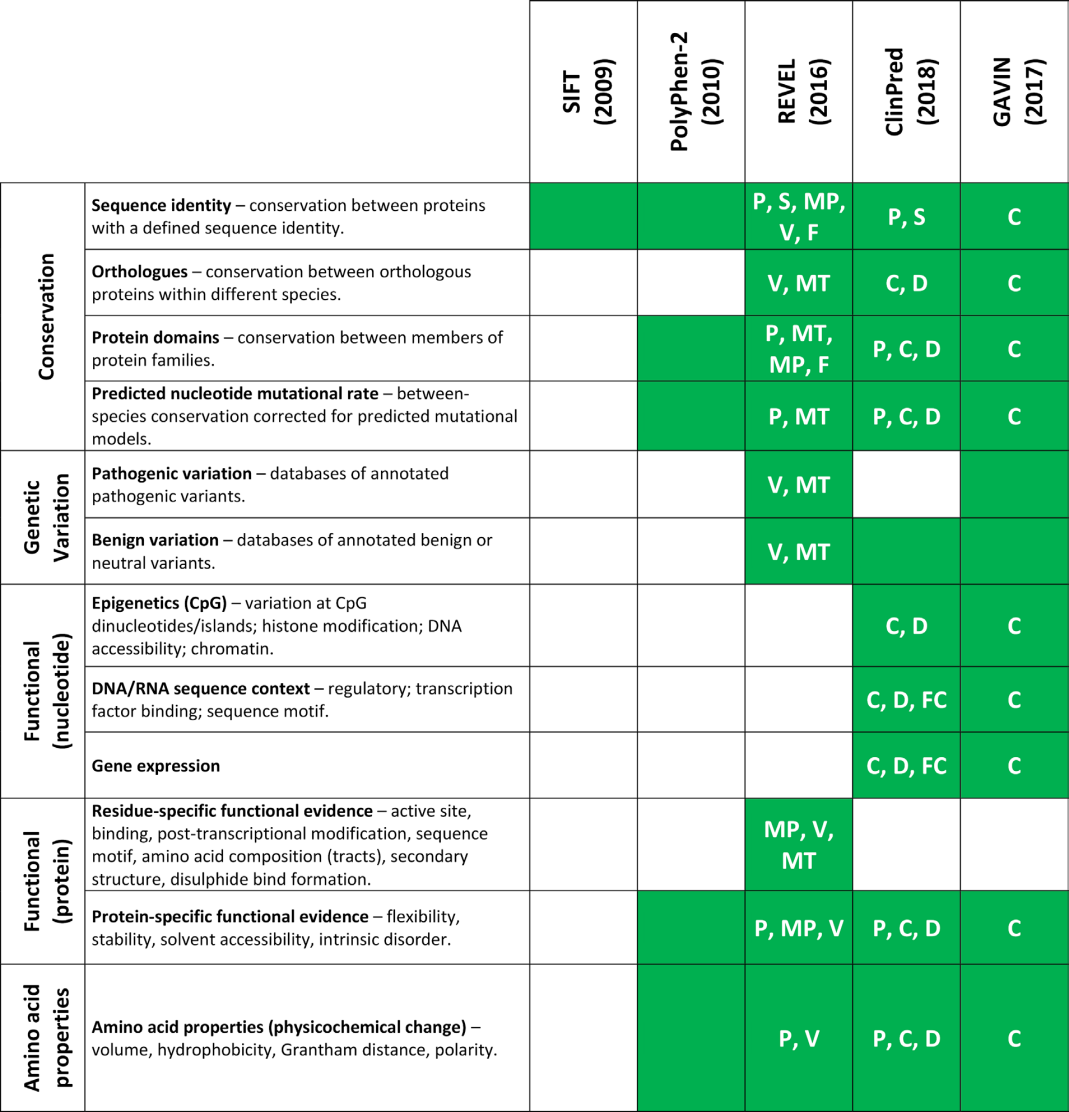
In silico pathogenicity predictor feature usage and source. Shading indicates that a category of evidence is used by the tool. Codes within each box indicate that the feature is inherited from another tool. Feature lists were taken from the tools' original publications, supplementary materials and available online material. C, CADD; D, DANN; F, FATHMM; FC, FitCons; MP, MutPred; MT, MutationTaster; P, PolyPhen-2; S, SIFT; V, VEST. An extended version is shown in online supplementary figure S1.

We compared the feature list of all tools benchmarked in this study (PolyPhen-2, SIFT, REVEL, GAVIN and ClinPred) and, in the case of the meta-predictors, the tools that they use as part of their algorithm (MPC,^30^ MutPred,^31^ VEST,^32^ CADD,^33^ DANN,^34^ SNPEff,^35^ FATHMM,^36^ FitCons^37^ and MutationTaster^38^). Features were split into five broad categories: Conservation, Genetic variation, Functional evidence (nucleotide), Functional evidence (protein) and Amino acid properties (see figure 2 and online supplementary figure S1). In general, the meta-predictors employ a wider variety of sources and are less heavily reliant on conservation alone. CADD/DANN and FitCons, and by extension GAVIN and ClinPred, are the only predictors with features within the *Functional (nucleotide*) category and are therefore able to predict the pathogenicity of a variant in the context of its nucleotide change, regardless of whether there is a resultant amino acid change.

10.1136/jmedgenet-2020-107003.supp2 Supplementary data

### Benchmarking predictor performance in the open and clinical datasets

Initially, each of the tools was benchmarked according to the threshold provided by the tools’ authors. This analysis involved a dichotomisation of scores with no intermediate range (see table 1).

**Table 1:** Results of variant classification for individual tool, and two consensus-based combinations, for the (A) open (n=8480) and (B) clinical (n=1757) datasets

		True positive	True negative	False positive	False negative	Sensitivity	Specificity	MCC	LR+	LR−
(A) Open dataset										
Individual	SIFT	2302	3857	1878	443	0.84	0.67	0.48	2.6:1	1:4.2
	PolyPhen-2	2387	4177	1558	358	0.87	0.73	0.56	3.2:1	1:5.6
	REVEL	2394	5445	290	351	0.87	0.95	0.83	17.2:1	1:7.4
	GAVIN	2615	5611	124	130	0.95	0.98	0.93	44.1:1	1:20.7
	ClinPred	2469	5731	4	276	0.90	1.00	0.93	1289.6:1	1:9.9
Consensus	SIFT+PolyPhen-2	2240	3410	2325	505	0.82	0.59	0.39	2:1	1:3.2
	REVEL+ClinPred	2233	5442	293	512	0.81	0.95	0.78	15.9:1	1:5.1
(B) Clinical dataset										
Individual	SIFT	1031	212	406	108	0.91	0.34	0.31	1.38:1	1:3.62
	PolyPhen-2	1021	211	407	118	0.90	0.34	0.29	1.36:1	1:3.3
	REVEL	983	370	248	156	0.86	0.60	0.48	2.15:1	1:4.37
	GAVIN	1100	157	461	39	0.97	0.25	0.33	1.29:1	1:7.42
	ClinPred	1107	167	451	32	0.97	0.27	0.36	1.33:1	1:9.62
Consensus	SIFT+PolyPhen-2	960	135	483	179	0.84	0.22	0.08	1.08:1	1:1.39
	REVEL+ClinPred	973	142	476	166	0.85	0.23	0.11	1.11:1	1:1.58

For consensus-based results, non-concordant, where tools disagree on the classification, were considered incorrect. Matthews correlation coefficient (MCC) was calculated as follows:

LR+ is the positive likelihood ratio; LR− is the negative likelihood ratio.

FN, false negatives (ie, pathogenic variants predicted to be benign); FP, false positives (ie, benign variants predicted to be pathogenic); TN, true negatives (ie, benign variants predicted to be benign); TP, true positives (ie, pathogenic variants predicted to be pathogenic).

**Figure 3:**
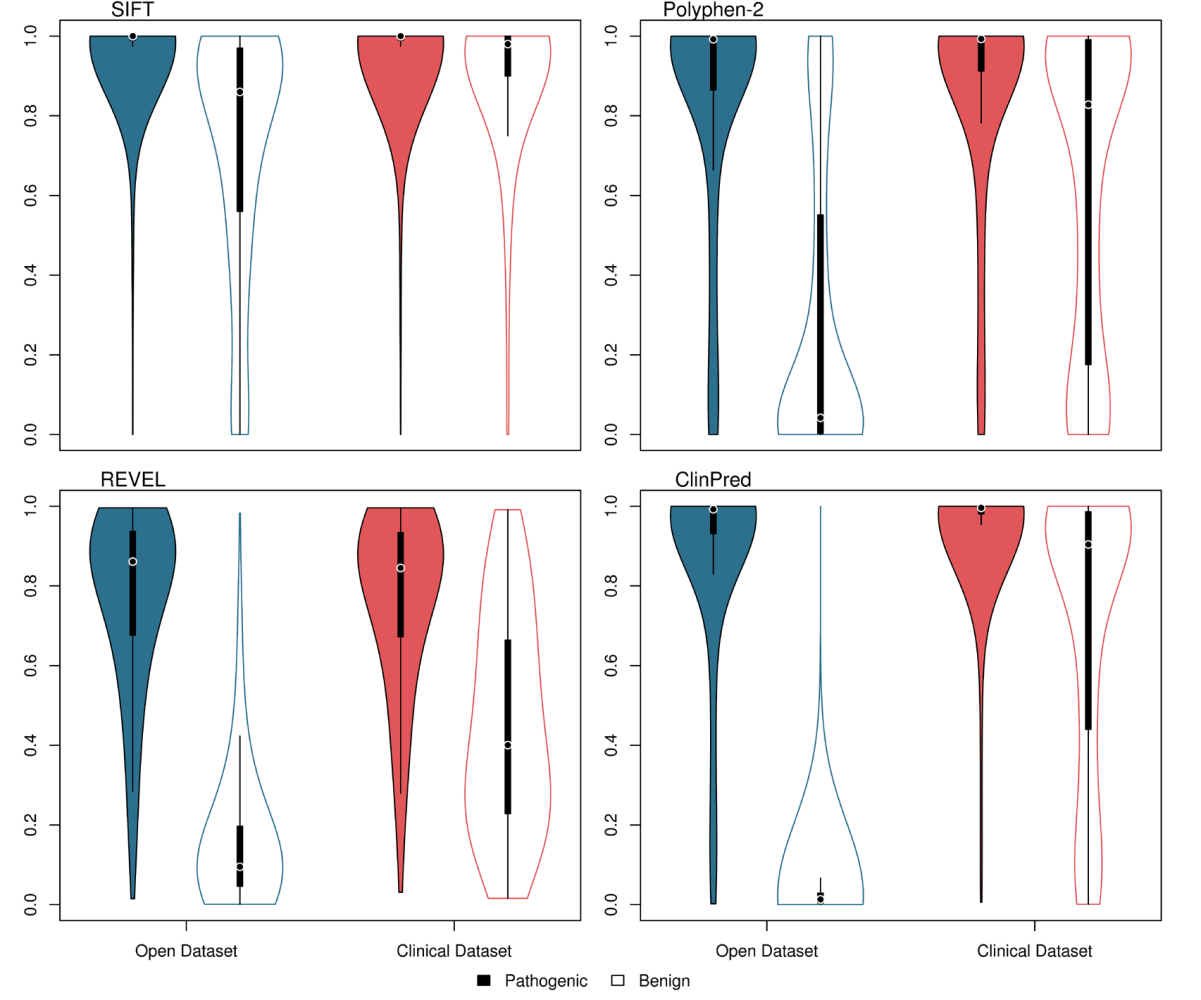
Violin plot showing variant scores for SIFT, PolyPhen-2, REVEL and ClinPred using two datasets. Open dataset—blue; clinical dataset—red; pathogenic variants—filled; benign variants—unfilled. Plot was generated in *R* using the 'vioplot' function in the 'vioplot' library. For ease of comparison, SIFT scores have been inverted.

**Figure 4:**
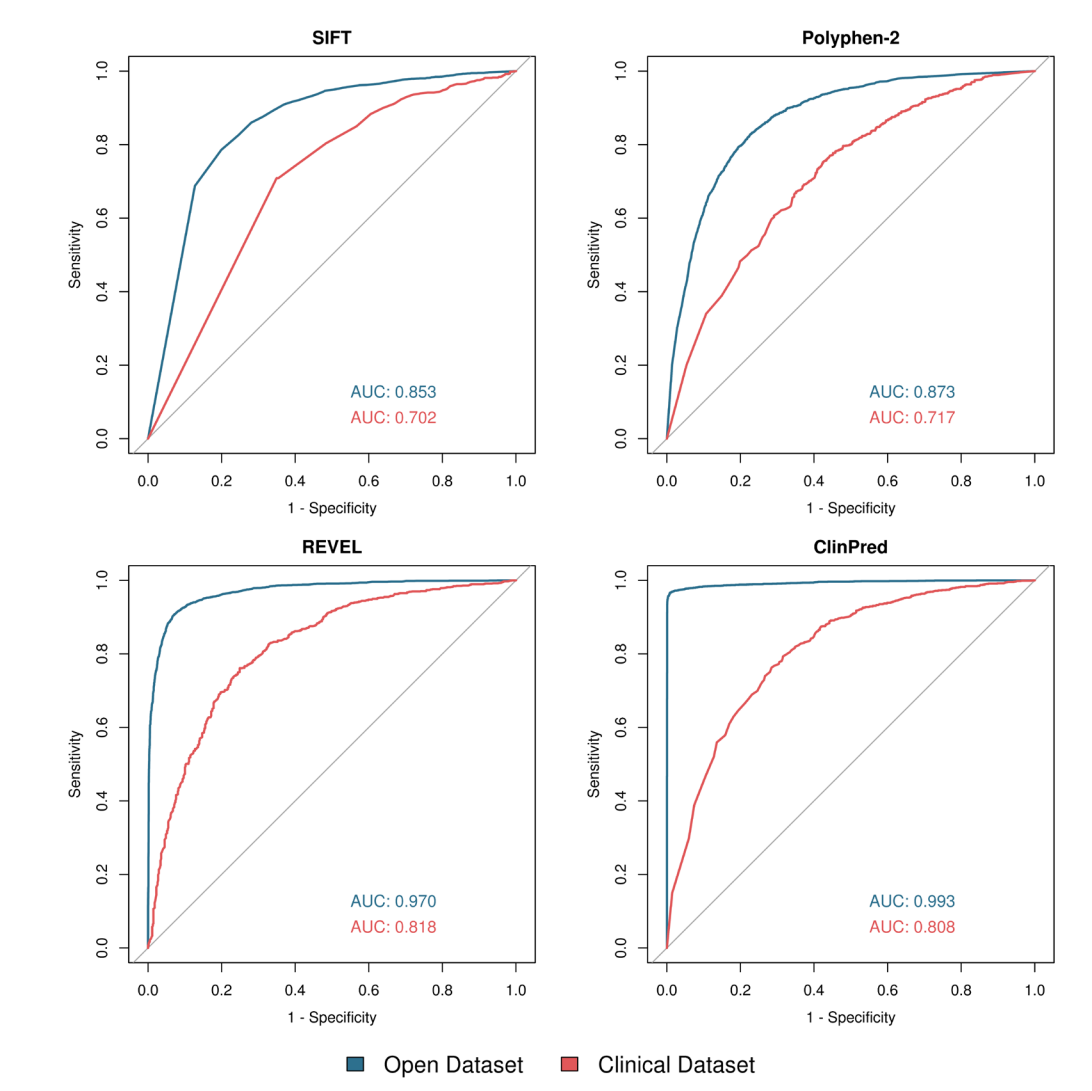
Receiver operating characteristic (ROC) curves for SIFT, PolyPhen-2, REVEL and ClinPred using two datasets. Open dataset—blue; clinical dataset—red. Generated in *R* using the ‘roc’ and ‘plot.roc’ functions in the ‘pROC’ library. Area under the ROC curve (AUC) was calculated in R using the ‘roc’ function. For ease of comparison, SIFT scores have been inverted.

The distribution of scores from SIFT, PolyPhen-2, REVEL and ClinPred is shown in figure 3 and receiver operating characteristic (ROC) curves are shown in figure 4. Of the tools with numerical outputs, ClinPred has the highest discriminatory power for the open dataset with an area under the ROC curve (AUC) of 0.993, while REVEL has the highest AUC for the clinical dataset of 0.818. The two meta-predictors outperformed SIFT and PolyPhen-2 in both datasets. In agreement with tool author benchmarking,^12–14^ the meta-predictors REVEL, ClinPred and GAVIN were highly proficient at classifying the variants in the open dataset, achieving sensitivities of 0.87, 0.90 and 0.95, and specificities of 0.95, 1.00 and 0.98, respectively. For variants in the clinical dataset, although the sensitivity of each tool remained largely constant, the specificity of all tools dropped considerably. For REVEL, ClinPred and GAVIN, specificity is reduced to 0.60, 0.27 and 0.25, respectively (table 1).

It was apparent that the threshold suggested by the tools’ authors was not well suited to both datasets, given the tools’ high sensitivity but low specificity in the clinical dataset. In order to correct for this, we performed a supplementary analysis for those predictors which gave a numerical output (SIFT, PolyPhen-2, REVEL and ClinPred). Here, a variable threshold was allowed for each tool to give a common sensitivity of 0.9 (ie, pathogenic variation is called correctly 90% of the time). The threshold required to give a sensitivity of 0.9 in each tool is shown in online supplementary table S2. The specificity of each tool at the determined threshold is shown in online supplementary figure S2. When allowed a variable threshold, the tools’ specificity increased significantly, with PolyPhen-2, SIFT, REVEL and ClinPred achieving a specificity of 0.67, 0.63, 0.93 and 0.99 for the open dataset, and 0.34, 0.33, 0.52 and 0.51 for the clinical dataset, respectively. In order to include GAVIN in this analysis, a third analysis was performed in which each tool was given a threshold to match the sensitivity achieved by GAVIN in each of the datasets. The specificity of all five tools is shown in online supplementary figure S3, and the sensitivity and threshold for each tool is shown in online supplementary table S3.

### Use of individual tools versus a consensus-based approach between multiple tools

**Figure 5:**
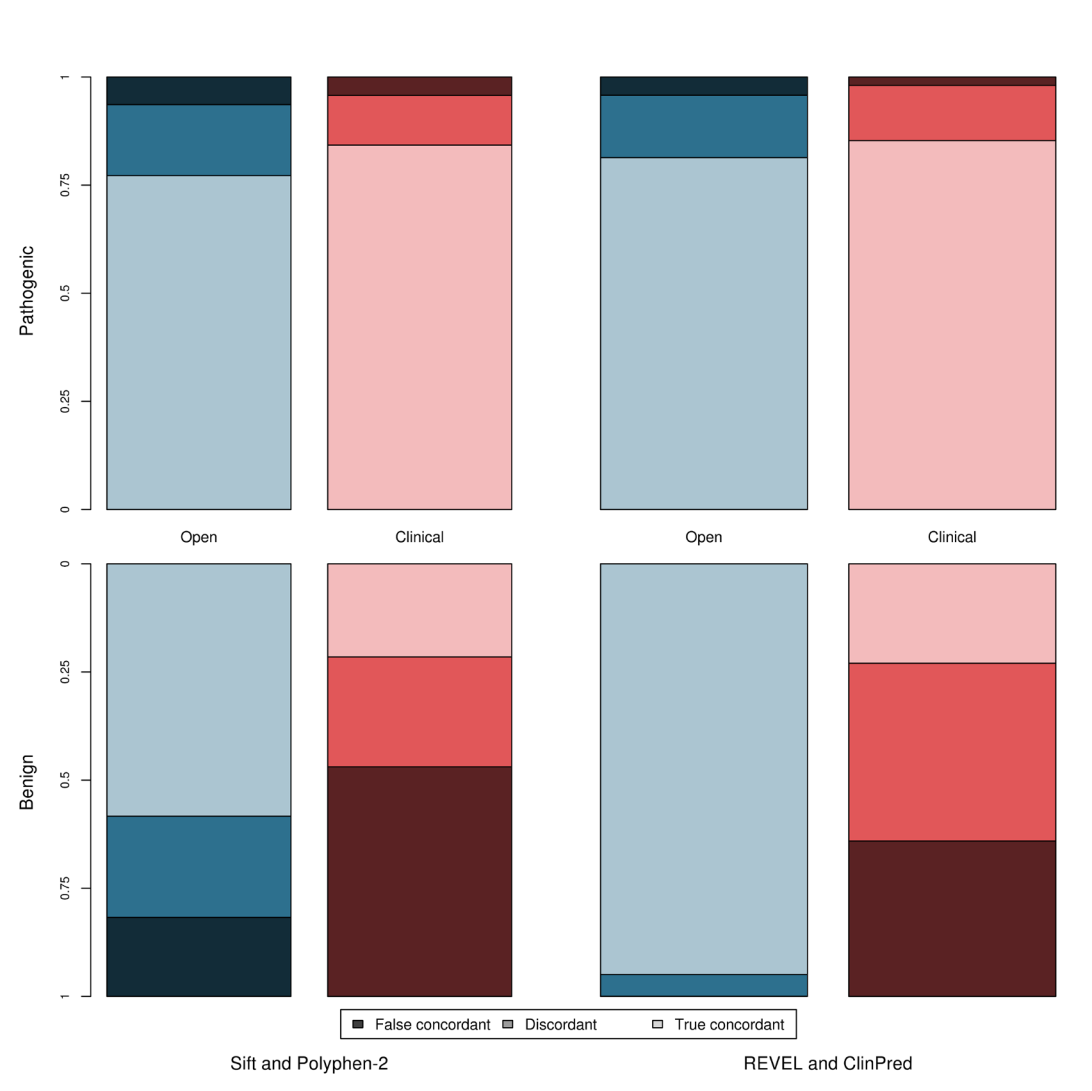
Concordance between tools separated by dataset and classification (pathogenic and benign). Open dataset—blue; clinical dataset—red; pathogenic variants—top graph; benign variants—bottom graph. True concordance indicates that the tools agree and were correct. False concordance indicates that the tools agree but were incorrect. Discordance indicates that the tools disagreed on the classification.

In accordance with current variant classification guidelines, we investigated the effect of performing a consensus-based analysis, using two commonly used tools, SIFT and PolyPhen-2, and two meta-predictors, REVEL and ClinPred, to determine whether this combined approach has improved sensitivity/specificity over the individual tools. Figure 5 shows the true concordance rate (correct classification by all tools), false concordance rate (incorrect classification by all tools) and discordance rate (disagreement between tools) for each of these tool pairings for the pathogenic and benign variants in both datasets. Within the clinically relevant dataset, the tools are either falsely concordant or discordant for ~15% of pathogenic variants but ~78% of benign variants. The sensitivity and specificity of this approach is shown in table 1. Use of a consensus-based approach may introduce a third ‘discordance’ category to the classification where tools disagree and no in silico evidence can be used, which applied to 21% and 16% of variants when considering the concordance of PolyPhen-2 and SIFT, and 8% and 23% when considering the concordance between REVEL and ClinPred, for the open and clinical datasets, respectively.

An alternative ‘majority rule’ method can instead be applied. Here, more than three tools are used, and the result agreed by >50% of tools selected. This method eliminates the ‘discordance’ category, as dissenting tools are ignored. Two majority-based analyses were performed using (1) all five tools (SIFT, Polyphen-2, REVEL, GAVIN and ClinPred) and (2) using only the meta-predictors (REVEL, GAVIN and ClinPred). The sensitivity and specificity of this majority-based approach is shown in online supplementary table S4. While this approach did improve on the strict concordance approach outlined previously, and is commonly applied in clinical genomics, the false concordance was still high and the highest specificity in the clinical dataset, achieved using a majority voting based approach with all five tools, was 0.32 (in contrast to the specificity of 0.60 achieved by REVEL in the same dataset).

## Discussion

We have compared the performance of five in silico pathogenicity predictors—two tools used routinely in variant classification (SIFT and PolyPhen-2) and three recently developed clinical meta-predictors (REVEL, ClinPred and GAVIN)—using two variant datasets: an open dataset collated using the selection strategy commonly employed when benchmarking tool performance, and a clinically representative dataset composed of rare and novel variants identified through high-throughput research and clinical sequencing with manual classification. Overall, the data herein show that meta-predictors have a greater sensitivity and specificity than the classic tools in both variant datasets. However, despite the increased accuracy of the meta-predictors, all tools performed substantially worse in the clinical dataset compared with the open dataset. This difference in tool performance illustrates the importance of considering the provenance of variants when benchmarking tools and how overfitting of a classifier to the training dataset can occur when increasingly large sets of variant features are used. The two datasets herein were constructed using very different methodologies, which determine the variants present within each. The open dataset, composed of variants derived from online repositories, is modelled on the methods commonly used when constructing test datasets. The tools performed universally well when characterising this dataset, indicating that these variants inherently possess features easily identifiable to the in silico predictors. In contrast, the clinical dataset is composed of variants identified through research and clinical next-generation sequencing pipelines, which had undergone multiple rounds of variant filtering and selection. Many variants within the open dataset would be automatically filtered out of the clinical dataset, based on MAF for example, and this dataset therefore gives a more representative assessment of the performance of such tools in genomic diagnostics laboratories—an assessment not previously performed.

Our analysis suggests that REVEL performs best when classifying rare variants routinely identified in clinical sequencing pipelines, with an AUC for our clinical dataset of 0.818, followed closely by ClinPred with an AUC of 0.808 (figure 4) and with a higher specificity than GAVIN in a direct (although suboptimal) comparison (online supplementary figure S3). While the REVEL team does not suggest a strict threshold for categorisation, in our analysis for the clinical dataset, a threshold of 0.43 gave a sensitivity of 0.9, and a specificity of 0.52, which is comparable with previous studies’ threshold of 0.5.^14^

Current guidelines on the classification of variants indicate that evidence should only apply when multiple tools are concordant.^1^ However, the use of concordance may introduce a third category to variants classification (discordance), where there is disagreement between tools and therefore the tools cannot be used as evidence to categorise the variant as either benign or pathogenic. The use of a majority-voting system appears to improve performance over a strict concordance approach, but our data show that both concordance methodologies give a lower sensitivity and specificity than the use of either of these tools in isolation, and furthermore that their performance is below that of the meta-predictors.

As with all similar studies, we were limited by the availability of novel variants absent from online databases such as gnomAD. The use of under-represented and genetically isolated populations, such as the Amish, allowed for the identification of several novel benign variants and suggests that such populations may be a rich source for future studies. We also identified several both pathogenic and benign variants in a clinical population through a translational research study (DDD). While steps were taken to ensure that the benign variants attained from this group were indeed benign (all variants were present within either monoallelic genes or in biallelic genes in a homozygous state, and were annotated by the referring clinician as having no contribution towards the patient’s clinical phenotype), nonetheless it cannot be guaranteed that the variants had no impact of protein function. The study underlines the need for improved data-sharing between clinical laboratories, including both pathogenic and benign rare variants.

This study supports the adoption of in silico meta-predictors for use in variant classification but recommends the use of a single meta-predictor over a consensus-based approach, as recommended by current ACMG guidelines.^1^ Each of the tools uses different though heavily overlapping data sources and the feature list used by a tool should be carefully considered before the tool is used. Our results also suggest that tools that use gnomAD data directly may have low specificity when classifying rare or novel variants and that care should be taken when using these tools in conjunction with the ACGS guidelines, as presence in or absence from the gnomAD database is already accounted for in other evidence criteria. Although use of a meta-predictor tool offers advantages over the use of previously available and widely adopted in silico tools, there remain issues to be addressed before they can be used at a level greater than supporting evidence for clinical variant interpretation.

## Data Availability

Data are available in a public, open access repository. All data relevant to the study are included in the article or uploaded as online supplementary information. The clinical dataset (online supplemental table S1) is released under the CC-BY license.

## References

[1] Richards S , Aziz N , Bale S , Bick D , Das S , Gastier-Foster J , Grody WW , Hegde M , Lyon E , Spector E , Voelkerding K , Rehm HL , ACMG Laboratory Quality Assurance Committee . Standards and guidelines for the interpretation of sequence variants: a joint consensus recommendation of the American College of Medical Genetics and Genomics and the Association for Molecular Pathology. Genet Med 2015;17:405–23. 10.1038/gim.2015.30 25741868 PMC4544753

[2] Sim N-L , Kumar P , Hu J , Henikoff S , Schneider G , Ng PC . SIFT web server: predicting effects of amino acid substitutions on proteins. Nucleic Acids Res 2012;40:W452–7. 10.1093/nar/gks539 22689647 PMC3394338

[3] Thusberg J , Olatubosun A , Vihinen M . Performance of mutation pathogenicity prediction methods on missense variants. Hum Mutat 2011;32:358–68. 10.1002/humu.21445 21412949

[4] Ellard S , Baple E , Berry I , Forrester N , Turnbull C , Owens M . ACGS best practice guidelines for variant classification; 2019. https://www.acgs.uk.com/news/acgs-best-practice-guidelines-for-variant-classification-2019/

[5] Grantham R . Amino acid difference formula to help explain protein evolution. Science 1974;185:862–4. 10.1126/science.185.4154.862 4843792

[6] Dayhoff MO , Schwartz RM , Orcutt BC . A model of evolutionary change in proteins. In: Atlas of protein sequence and structure, 1978: 345–52.

[7] Adzhubei IA , Schmidt S , Peshkin L , Ramensky VE , Gerasimova A , Bork P , Kondrashov AS , Sunyaev SR . A method and server for predicting damaging missense mutations. Nat Methods 2010;7:248–9. 10.1038/nmeth0410-248 20354512 PMC2855889

[8] ENCODE Project Consortium . An integrated encyclopedia of DNA elements in the human genome. Nature 2012;489:57–74. 10.1038/nature11247 22955616 PMC3439153

[9] Karczewski KJ , Francioli LC , Tiao G , Cummings BB , Alföldi J , Wang Q , Collins RL , Laricchia KM , Ganna A , Birnbaum DP , Gauthier LD , Brand H , Solomonson M , Watts NA , Rhodes D , Singer-Berk M , Seaby EG , Kosmicki JA , Walters RK , Tashman K , Farjoun Y , Banks E , Poterba T , Wang A , Seed C , Whiffin N , Chong JX , Samocha KE , Pierce-Hoffman E , Zappala Z , O’Donnell-Luria AH , Minikel EV , Weisburd B , Lek M , Ware JS , Vittal C , Armean IM , Bergelson L , Cibulskis K , Connolly KM , Covarrubias M , Donnelly S , Ferriera S , Gabriel S , Gentry J , Gupta N , Jeandet T , Kaplan D , Llanwarne C , Munshi R , Novod S , Petrillo N , Roazen D , Ruano-Rubio V , Saltzman A , Schleicher M , Soto J , Tibbetts K , Tolonen C , Wade G , Talkowski ME , Neale BM , Daly MJ , MacArthur DG , Consortium TGAD . Variation across 141 456 human exomes and genomes reveals the spectrum of loss-of-function intolerance across human protein-coding genes. bioRxiv 2019:531210.

[10] Landrum MJ , Lee JM , Benson M , Brown GR , Chao C , Chitipiralla S , Gu B , Hart J , Hoffman D , Jang W , Karapetyan K , Katz K , Liu C , Maddipatla Z , Malheiro A , McDaniel K , Ovetsky M , Riley G , Zhou G , Holmes JB , Kattman BL , Maglott DR . ClinVar: improving access to variant interpretations and supporting evidence. Nucleic Acids Res 2018;46:D1062–7. 10.1093/nar/gkx1153 29165669 PMC5753237

[11] Stenson PD , Mort M , Ball EV , Shaw K , Phillips A , Cooper DN . The human gene mutation database: building a comprehensive mutation repository for clinical and molecular genetics, diagnostic testing and personalized genomic medicine. Hum Genet 2014;133:1–9. 10.1007/s00439-013-1358-4 24077912 PMC3898141

[12] Ioannidis NM , Rothstein JH , Pejaver V , Middha S , McDonnell SK , Baheti S , Musolf A , Li Q , Holzinger E , Karyadi D , Cannon-Albright LA , Teerlink CC , Stanford JL , Isaacs WB , Xu J , Cooney KA , Lange EM , Schleutker J , Carpten JD , Powell IJ , Cussenot O , Cancel-Tassin G , Giles GG , MacInnis RJ , Maier C , Hsieh C-L , Wiklund F , Catalona WJ , Foulkes WD , Mandal D , Eeles RA , Kote-Jarai Z , Bustamante CD , Schaid DJ , Hastie T , Ostrander EA , Bailey-Wilson JE , Radivojac P , Thibodeau SN , Whittemore AS , Sieh W . REVEL: an ensemble method for predicting the pathogenicity of rare missense variants. Am J Hum Genet 2016;99:877–85. 10.1016/j.ajhg.2016.08.016 27666373 PMC5065685

[13] van der Velde KJ , de Boer EN , van Diemen CC , Sikkema-Raddatz B , Abbott KM , Knopperts A , Franke L , Sijmons RH , de Koning TJ , Wijmenga C , Sinke RJ , Swertz MA . GAVIN: Gene-Aware variant interpretation for medical sequencing. Genome Biol 2017;18:6. 10.1186/s13059-016-1141-7 28093075 PMC5240400

[14] Alirezaie N , Kernohan KD , Hartley T , Majewski J , Hocking TD . ClinPred: prediction tool to identify disease-relevant nonsynonymous single-nucleotide variants. Am J Hum Genet 2018;103:474–83. 10.1016/j.ajhg.2018.08.005 30220433 PMC6174354

[15] Subramanian J , Simon R . Overfitting in prediction models—is it a problem only in high dimensions? Contemp Clin Trials 2013;36:636–41. 10.1016/j.cct.2013.06.011 23811117

[16] Hawkins DM . The problem of overfitting. J Chem Inf Comput Sci 2004;44:1–12. 10.1021/ci0342472 14741005

[17] Karczewski KJ , Francioli LC , Tiao G , Cummings BB , Alföldi J , Wang Q , Collins RL , Laricchia KM , Ganna A , Birnbaum DP , Gauthier LD , Brand H , Solomonson M , Watts NA , Rhodes D , Singer-Berk M , England EM , Seaby EG , Kosmicki JA , Walters RK , Tashman K , Farjoun Y , Banks E , Poterba T , Wang A , Seed C , Whiffin N , Chong JX , Samocha KE , Pierce-Hoffman E , Zappala Z , O'Donnell-Luria AH , Minikel EV , Weisburd B , Lek M , Ware JS , Vittal C , Armean IM , Bergelson L , Cibulskis K , Connolly KM , Covarrubias M , Donnelly S , Ferriera S , Gabriel S , Gentry J , Gupta N , Jeandet T , Kaplan D , Llanwarne C , Munshi R , Novod S , Petrillo N , Roazen D , Ruano-Rubio V , Saltzman A , Schleicher M , Soto J , Tibbetts K , Tolonen C , Wade G , Talkowski ME , Neale BM , Daly MJ , MacArthur DG , Genome Aggregation Database Consortium . The mutational constraint spectrum quantified from variation in 141,456 humans. Nature 2020;581:434–43. 10.1038/s41586-020-2308-7 32461654 PMC7334197

[18] Ghosh R , Oak N , Plon SE . Evaluation of in silico algorithms for use with ACMG/AMP clinical variant interpretation guidelines. Genome Biol 2017;18:225. 10.1186/s13059-017-1353-5 29179779 PMC5704597

[19] Sasidharan Nair P , Vihinen M . VariBench: a benchmark database for variations. Hum Mutat 2013;34:42–9. 10.1002/humu.22204 22903802

[20] Grimm DG , Azencott C-A , Aicheler F , Gieraths U , MacArthur DG , Samocha KE , Cooper DN , Stenson PD , Daly MJ , Smoller JW , Duncan LE , Borgwardt KM . The evaluation of tools used to predict the impact of missense variants is hindered by two types of circularity. Hum Mutat 2015;36:513–23. 10.1002/humu.22768 25684150 PMC4409520

[21] UniProt Consortium . UniProt: a worldwide hub of protein knowledge. Nucleic Acids Res 2019;47:D506–15. 10.1093/nar/gky1049 30395287 PMC6323992

[22] Dong C , Wei P , Jian X , Gibbs R , Boerwinkle E , Wang K , Liu X . Comparison and integration of deleteriousness prediction methods for nonsynonymous SNVs in whole exome sequencing studies. Hum Mol Genet 2015;24:2125–37. 10.1093/hmg/ddu733 25552646 PMC4375422

[23] Niroula A , Vihinen M . How good are pathogenicity predictors in detecting benign variants? PLoS Comput Biol 2019;15:e1006481. 10.1371/journal.pcbi.1006481 30742610 PMC6386394

[24] Wright CF , Fitzgerald TW , Jones WD , Clayton S , McRae JF , van Kogelenberg M , King DA , Ambridge K , Barrett DM , Bayzetinova T , Bevan AP , Bragin E , Chatzimichali EA , Gribble S , Jones P , Krishnappa N , Mason LE , Miller R , Morley KI , Parthiban V , Prigmore E , Rajan D , Sifrim A , Swaminathan GJ , Tivey AR , Middleton A , Parker M , Carter NP , Barrett JC , Hurles ME , FitzPatrick DR , Firth HV , DDD study . Genetic diagnosis of developmental disorders in the DDD study: a scalable analysis of genome-wide research data. Lancet 2015;385:1305–14. 10.1016/S0140-6736(14)61705-0 25529582 PMC4392068

[25] Stenson PD , Mort M , Ball EV , Evans K , Hayden M , Heywood S , Hussain M , Phillips AD , Cooper DN . The human gene mutation database: towards a comprehensive repository of inherited mutation data for medical research, genetic diagnosis and next-generation sequencing studies. Hum Genet 2017;136:665–77. 10.1007/s00439-017-1779-6 28349240 PMC5429360

[26] Hamosh A , Scott AF , Amberger JS , Bocchini CA , McKusick VA . Online Mendelian inheritance in man (OMIM), a knowledgebase of human genes and genetic disorders. Nucleic Acids Res 2005;33:D514–7. 10.1093/nar/gki033 15608251 PMC539987

[27] Wright CF , McRae JF , Clayton S , Gallone G , Aitken S , FitzGerald TW , Jones P , Prigmore E , Rajan D , Lord J , Sifrim A , Kelsell R , Parker MJ , Barrett JC , Hurles ME , FitzPatrick DR , Firth HV , DDD Study . Making new genetic diagnoses with old data: iterative reanalysis and reporting from genome-wide data in 1,133 families with developmental disorders. Genet Med 2018;20:1216–23. 10.1038/gim.2017.246 29323667 PMC5912505

[28] Thormann A , Halachev M , McLaren W , Moore DJ , Svinti V , Campbell A , Kerr SM , Tischkowitz M , Hunt SE , Dunlop MG , Hurles ME , Wright CF , Firth HV , Cunningham F , FitzPatrick DR . Flexible and scalable diagnostic filtering of genomic variants using G2P with Ensembl VEP. Nat Commun 2019;10:2373. 10.1038/s41467-019-10016-3 31147538 PMC6542828

[29] McLaren W , Gil L , Hunt SE , Riat HS , Ritchie GRS , Thormann A , Flicek P , Cunningham F . The Ensembl variant effect predictor. Genome Biol 2016;17:122. 10.1186/s13059-016-0974-4 27268795 PMC4893825

[30] Samocha KE , Kosmicki JA , Karczewski KJ , O’Donnell-Luria AH , Pierce-Hoffman E , MacArthur DG , Neale BM , Daly MJ . Regional missense constraint improves variant deleteriousness prediction. bioRxiv 2017;148353.

[31] Li B , Krishnan VG , Mort ME , Xin F , Kamati KK , Cooper DN , Mooney SD , Radivojac P . Automated inference of molecular mechanisms of disease from amino acid substitutions. Bioinformatics 2009;25:2744–50. 10.1093/bioinformatics/btp528 19734154 PMC3140805

[32] Carter H , Douville C , Stenson PD , Cooper DN , Karchin R . Identifying Mendelian disease genes with the variant effect scoring tool. BMC Genomics 2013;14(Suppl 3):S3. 10.1186/1471-2164-14-S3-S3 PMC3665549 23819870

[33] Kircher M , Witten DM , Jain P , O'Roak BJ , Cooper GM , Shendure J . A general framework for estimating the relative pathogenicity of human genetic variants. Nat Genet 2014;46:310–5. 10.1038/ng.2892 24487276 PMC3992975

[34] Quang D , Chen Y , Xie X . DANN: a deep learning approach for annotating the pathogenicity of genetic variants. Bioinformatics 2015;31:761–3. 10.1093/bioinformatics/btu703 25338716 PMC4341060

[35] Cingolani P , Platts A , Wang LL , Coon M , Nguyen T , Wang L , Land SJ , Lu X , Ruden DM . A program for annotating and predicting the effects of single nucleotide polymorphisms, SnpEff: SNPs in the genome of Drosophila melanogaster strain w1118; iso-2; iso-3. Fly 2012;6:80–92. 10.4161/fly.19695 22728672 PMC3679285

[36] Shihab HA , Gough J , Cooper DN , Stenson PD , Barker GLA , Edwards KJ , Day INM , Gaunt TR . Predicting the functional, molecular, and phenotypic consequences of amino acid substitutions using hidden Markov models. Hum Mutat 2013;34:57–65. 10.1002/humu.22225 23033316 PMC3558800

[37] Gulko B , Hubisz MJ , Gronau I , Siepel A . A method for calculating probabilities of fitness consequences for point mutations across the human genome. Nat Genet 2015;47:276–83. 10.1038/ng.3196 25599402 PMC4342276

[38] Schwarz JM , Rödelsperger C , Schuelke M , Seelow D . MutationTaster evaluates disease-causing potential of sequence alterations. Nat Methods 2010;7:575–6. 10.1038/nmeth0810-575 20676075

