# Supplementary material for: Assessing performance of pathogenicity predictors using clinically relevant variant datasets
Source: J Med Genet. 2020 Aug 25;58(8):547–55. doi: 10.1136/jmedgenet-2020-107003 (PMC8327323; doi:10.1136/jmedgenet-2020-107003)
Supplement: Supplementary data [file jmedgenet-2020-107003supp002.pdf]

Supplemental Materials

Figure S1 - In silico pathogenicity predictor feature usage and source (extended).

Figure S2 - The specificity of SIFT, PolyPhen-2, REVEL and ClinPred for the Open (Blue) and Clinical (Red) datasets, with sensitivity set to 0.9.

Table S2 - Threshold required to give an approximate sensitivity of 0.9.

Figure S3 - The specificity of SIFT, Polyphen-2, REVEL, ClinPred and GAVIN with sensitivity set to that of GAVIN

Table S3 - Threshold required to give a sensitivity identical to that of GAVIN.

Table S4 - Sensitivity and specificity of concordance-based approached in (A) open and (B) clinical datasets.

		SIFT (2009)	PolyPhen-2 (2010)	REVEL (2016)	ClinPred (2018)	GAVIN (2017)	MutPred (2009)	MutationTaster (2010)	FATHMM (2013)	VEST (2013)	CADD (2014) / DANN (2015)
**Conservation**	**Sequence identity** – conservation between proteins with a defined sequence identity.			P, S, MP, V, F	P, S	C					P, S
	**Orthologues** – conservation between orthologous proteins within different species.			V, MT	C, D	C					
	**Protein domains** – conservation between members of protein families.			P, MT, MP, F	P, C, D	C					P
	**Predicted nucleotide mutational rate** – between-species conservation corrected for predicted mutational models.			P, MP	P, C, D	C					P
**Genetic Variation**	**Pathogenic variation** – databases of annotated pathogenic variants.			V, MT							
	**Benign variation** – databases of annotated benign or neutral variants.			V, MT							
**Functional (nucleotide)**	**Epigenetics (CpG)** – variation at CpG dinucleotides/islands; histone modification; DNA accessibility; chromatin.				C, D	C					
	**DNA/RNA sequence context** – regulatory; transcription factor binding; sequence motif.				C, D, FC	C					
	**Gene expression**				C, D, FC	C					
**Functional (protein)**	**Residue-specific functional evidence** – active site, binding, post-transcriptional modification, sequence motif, amino acid composition (tracts), secondary structure, disulphide bind formation.			MP, V, MT							
	**Protein-specific functional evidence** – flexibility, stability, solvent accessibility, intrinsic disorder.			P, MP, V	P, C, D	C					P
**Amino Acid Properties**	**Amino acid properties (physicochemical change)** – volume, hydrophobicity, Grantham distance, polarity.			P, V	P, C, D	C		P, C, D		P	P

Figure S1. *In silico* pathogenicity predictor feature usage and source (extended). Extended version of Figure 1 to include additional tools utilised by meta-predictors. Shading indicates that a category of evidence is utilised by the tool. Codes within each box indicate that the feature is inherited from another tool. Feature lists were taken from the tools' original publications, supplementary materials and available online material. P – PolyPhen-2; S – SIFT; MP – MutPred; V – VEST; C – CADD; D – DANN; MT – MutationTaster; F – FATHMM, FC - FitCons.

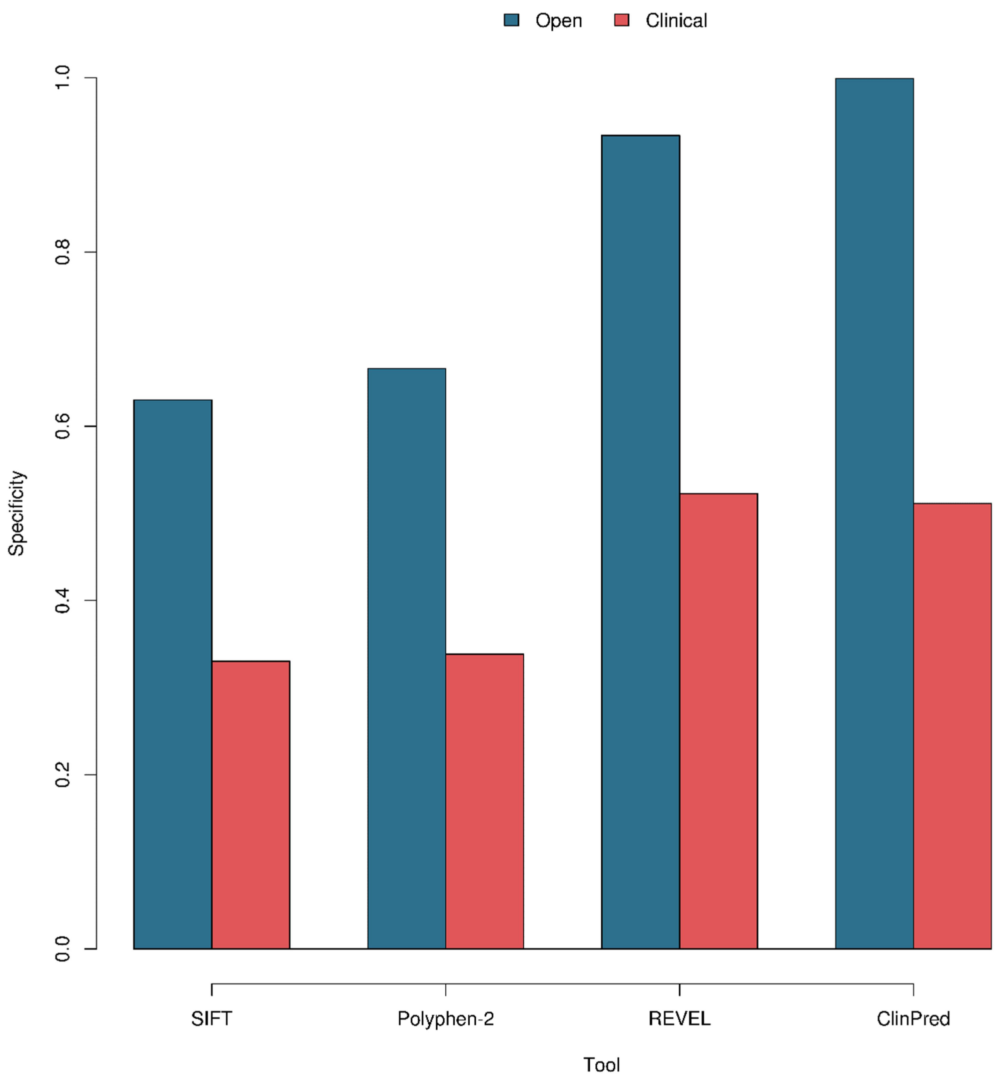

Figure S2. The specificity of SIFT, PolyPhen-2, REVEL and ClinPred for the Open (Blue) and Clinical (Red) datasets, with sensitivity set to 0.9. Thresholds for each tool required to give sensitivity of 0.9 are shown in Table S2.

	Dataset	
	Open	Clinical
SIFT	≤0.06	≤0.05
Polyphen	≥0.27	≥0.43
REVEL	≥0.44	≥0.43
ClinPred	≥0.50	≥0.91

Table S2. Threshold required to give a sensitivity of 0.9. The threshold that was applied for each of the tools to give a sensitivity of 0.9. The specificity achieved is shown in Figure S2.

Gunning AC, *et al. J Med Genet* 2021; 58:547–555. doi: 10.1136/jmedgenet-2020-107003

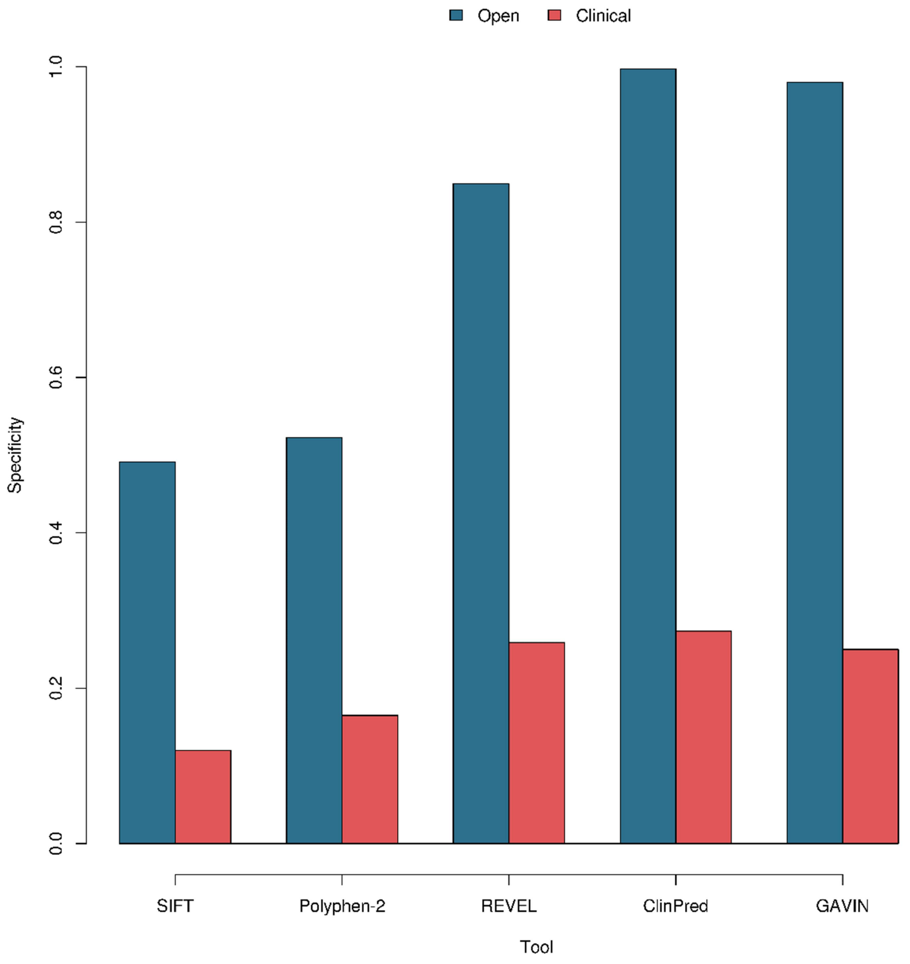

Figure S3. The specificity of SIFT, Polyphen-2, REVEL, ClinPred and GAVIN for the Open (Blue) and Clinical (Red) datasets, with sensitivity set to that of GAVIN (0.95 and 0.97 for the Open and Clinical datasets, respectively). Thresholds for each tool required are shown in Table S3.

	Dataset	
	Open	Clinical
SIFT	≤0.15	≤0.25
PolyPhen-2	≥0.054	≥0.052
REVEL	≥0.29	≥0.24
ClinPred	≥0.17	≥0.52

Table S3. Threshold required to give a sensitivity identical to that of GAVIN. 0.95 and 0.97 for the Open and Clinical datasets, respectively.

Gunning AC, *et al. J Med Genet* 2021; 58:547–555. doi: 10.1136/jmedgenet-2020-107003

A

Method	Open Dataset							
	TP	TN	FP	FN	Sensitivity	Specificity	LR+	LR-
(I) SIFT+Polyphen2	2240	3410	2325	505	0.82	0.59	2:1	1:3.2
(II) REVEL+ClinPred	2233	5442	293	512	0.81	0.95	15.9:1	1:5.1
(III) Majority (all tools)	2559	5475	260	186	0.93	0.95	20.6:1	1:14.1
(IV) Majority (meta-predictors)	2594	5687	48	151	0.94	0.99	112.9:1	1:18

B

Method	Clinical Dataset							
	TP	TN	FP	FN	Sensitivity	Specificity	LR+	LR-
(I) SIFT+Polyphen2	960	135	483	179	0.84	0.22	1.1:1	1:1.4
(II) REVEL+ClinPred	973	142	476	166	0.85	0.23	1.1:1	1:1.6
(III) Majority (all tools)	1098	193	425	41	0.96	0.31	1.4:1	1:8.7
(IV) Majority (meta-predictors)	1104	178	440	35	0.97	0.29	1.4:1	1:9.4

Table S4. Sensitivity and specificity of concordance-based approached in (A) open and (B) clinical datasets. Four approaches were applied: Strict concordance using pairs of tools, (I) SIFT and Polyphen-2 and (II) REVEL and ClinPred. A strict concordance was needed, where any discordance, or disagreement, between the tools was considered an incorrect call. Two majority voting approaches were also applied using (III) all 5 tools and (IV) the three meta-predictors REVEL, GAVIN and ClinPred. The majority voting approach had higher sensitivity, specificity and likelihood ratios than the pairwise approaches.

Gunning AC, *et al. J Med Genet* 2021; 58:547–555. doi: 10.1136/jmedgenet-2020-107003
